# A Rare Case of a Chylous Pleural Effusion as the Initial Manifestation of Synchronous Tumors

**DOI:** 10.7759/cureus.46903

**Published:** 2023-10-12

**Authors:** Mafalda Sequeira, Filipe Nogueira, Catarina Pestana Santos, Tiago Judas, Francisca Delerue

**Affiliations:** 1 Internal Medicine Service, Hospital Garcia de Orta, Almada, PRT; 2 Pathological Anatomy Service, Hospital Garcia de Orta, Almada, PRT

**Keywords:** histopathology, multi-disciplinary approach, invasive lobular breast carcinoma, diffuse b-cell non-hodgkin lymphoma, multiple primary malignancies

## Abstract

Multiple primary malignancies (MPMs) are defined as two or more histopathologically distinct malignancies in the same individual. MPMs are classified as synchronous when tumors are diagnosed within six months of each other. The most common malignancies in MPMs are melanoma, breast, lung, and prostate cancer. Synchronous lymphoma and solid tumors are relatively rare. In these cases, a multi-disciplinary approach to treatment is essential. The early detection of additional primary malignancies such as myeloid and lymphatic tumors will enable prompt management with curative intent. The authors present a case of diffuse B-cell non-Hodgkin lymphoma and invasive lobular breast carcinoma presented as a chylous pleural effusion.

## Introduction

Multiple primary malignancies (MPMs) have been increasingly recognized in recent decades, with a described incidence of 0.52%-11.7% in different countries. Digestive system tumors and adenocarcinomas were the most frequent ones [1]. The simultaneous diagnosis of lymphoma and solid tumors is still a relatively rare scenario. A chylous pleural effusion, as the initial presentation of MPMs, is seldom described [2]. The most common cause of chylothorax is neoplasm, which is responsible for more than 50% of cases. The main physiological mechanisms of pleural chylous effusion formation are compression/invasion of the thoracic duct and obliteration of the lymphatics after radiation therapy [3,4].

## Case presentation

The authors present the case of a 61-year-old female with a personal history of arterial hypertension, endovascular repair of thoracic aortic aneurysm, dyslipidemia, and renal nephrectomy due to complicated nephrolithiasis.

**Figure 1:**
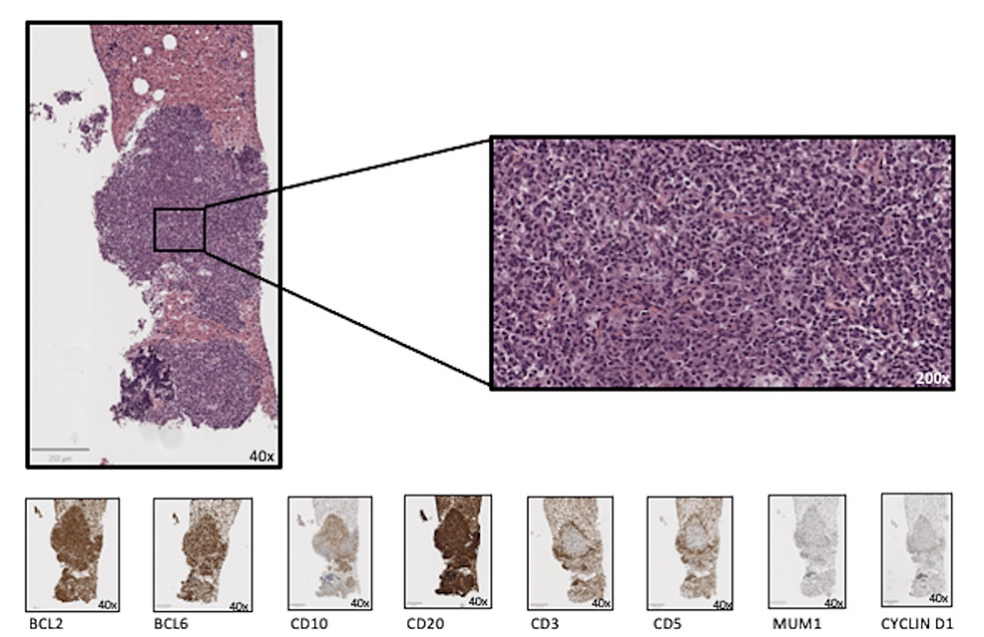
Cell block consisted of blood and lymphoid cells of medium and large size, isolated, and in aggregates. These cells are positive for BCL2, BCL6, CD10, and CD20, with Ki 67 of 80%, and negative for CD3, CD5, Cyclin D1, and MUM1. Compatible with the diagnosis of diffuse large B-cell lymphoma (germinal center type).

The patient was admitted to the internal medicine department due to complaints of progressive shortness of breath over the previous two months and right chest pain with pleuritic characteristics. She denied fever, cough, or weight loss. On physical examination, the patient was afebrile, normotensive, deep, and rapid breathing with signs of a right pleural effusion on pulmonary observation. Analytical evaluation was unrevealing. Chest radiography showed an extensive right pleural effusion. Diagnostic thoracentesis was performed, and chylous pleural fluid was obtained (triglycerides 150.9 mmol/L). Both microbiological and cytopathological examinations of the liquid were unremarkable. A pleural biopsy was also performed, which showed the presence of a marked non-specific lymphocytic inflammatory infiltrate, with no granulomas or neoplastic tissue. The hypothesis of lymphoproliferative disease was suspected, and a thoracic-abdominal-pelvic computerized tomography scan was performed that showed a moderate right pleural effusion, cutaneous thickening of the left breast with distortion of the parenchymal architecture in the upper-internal quadrant, and enlarged lymph nodes of the celiac territory. Celiac adenopathy aspiration cytology was obtained, through ultrasound endoscopy, with the histological result showing diffuse large B-cell lymphoma (germinal center type), which was confirmed by flux cytometry (Figure 1).

**Figure 2:**
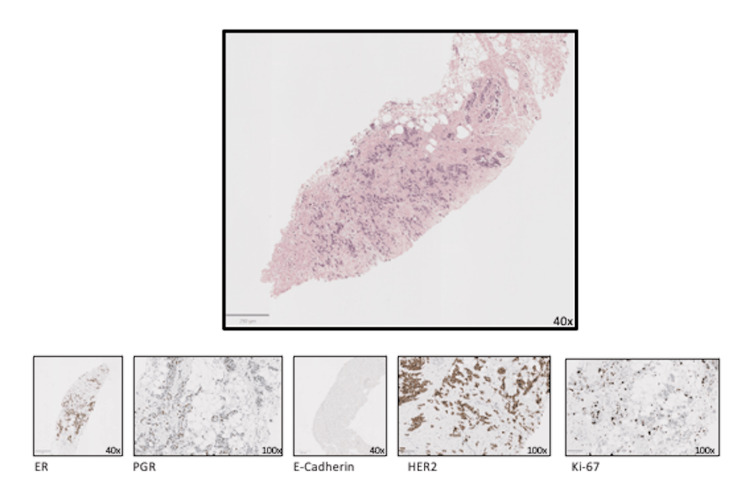
Mammary parenchyma with invasive lobular carcinoma, a classic and alveolar type, G2 (Ellis and Elston). Estrogen receptors (ER) - positive (100%); progesterone receptors (PGR) - positive (30%); CerbB2 - positive (3+); Ki 67 - 40%. E-cadherin - negative.

Ultrasound-guided biopsy of the breast lesion was performed and confirmed lobular carcinoma, an invasive, classic, and alveolar type (Figure 2).

For neoplasm staging, the following investigation was performed:

- Bone marrow aspirate with immunophenotyping, which was unrevealing.

- Positron emission tomography scan (PET scan) showed multiple secondary adenopathies of the mediastinum and upper abdomen and a suspicious metabolic image for neoplasm of the left breast. It also showed probable bilateral pleural metastization.

- MRI of the brain and spine that showed neoplastic involvement of the bone marrow, probably of lymphoproliferative nature. These lesions concerned the anterior and right lateral region of the vertebral body of D7, the anterior and right lateral of the vertebral body of D8, anterior of the vertebral body of D9, and the anterosuperior region of the vertebral body of D12.

At this point, the diagnosis of two synchronous multiple primary malignancies (MPMs), lymphoma and breast adenocarcinoma, was confirmed.

A chemotherapy regimen with rituximab, cyclophosphamide, doxorubicin, vincristine, and prednisone (R-CHOP) was started directed to lymphoma and hormone therapy with letrozole directed to breast cancer. Lumbar puncture was also performed with administration of intrathecal chemotherapy with methotrexate and hydrocortisone.

The patient had an unfavorable clinical outcome, having developed refractory septic shock of pulmonary origin, requiring admission to the intensive care unit, invasive ventilatory support, and dialysis support, having eventually died.

## Discussion

Patients with primary multiple malignancies are progressively increasing due to the prolonged survival of cancer patients and to the medical advances in diagnostic and therapies. The reported incidence of MPMTs has ranged from 0.52% to 11.7% in various studies from different countries. The most frequent sites of localization and pathology of tumors were the digestive system and adenocarcinomas, respectively [5,6].

Published studies and clinical experience prove that the increased incidence of multiple malignant tumors is a real challenge to the clinician, and, for this reason, it is often underdiagnosed. Despite the few cases published in the literature, from what we know about this disease, early diagnosis is essential, especially for the early initiation of aggressive and targeted therapy.

MPM pathophysiology is not well-understood; however, it is believed that their occurrence is influenced by a series of factors such as individual predisposition or by the action of carcinogenic factors acting on different organs at different times. It is known, as for other types of tumors, that the combination of genetic and environmental factors is fundamental for the development of synchronous tumors, although it has not been concluded, which of the two factors has a greater contribution in the case of these tumors [7].

The diagnosis of MPMs can be challenging, and there are no guidelines regarding treatment, although we know that there are several treatment modalities, including surgery, chemotherapy, and radiation, that can be applied depending on the types of tumors in question. For the reasons mentioned and, as it is a disease with high mortality and morbidity, we must always maintain a high level of suspicion when approaching patients with complex signs and symptoms [8].

## Conclusions

Our case shows a less common presentation of synchronous MPMs, either by clinical presentation (chylous pleural effusion) or by the type of neoplasms involved. Being neoplasm the most frequent cause of chylothorax, the occurrence of two neoplasms concomitantly is extremely rare in this setting.

Despite the rapid clinical workup, diagnosis, and treatment, the patient had an unfavorable outcome due to severe infectious complications. This clinical outcome comes in line with the general prognosis of synchronous tumors that usually display shorter survival times than patients with single or metachronous tumors.

## References

[1] Testori A, Cioffi U, De Simone M (2015). Multiple primary synchronous malignant tumors. BMC Res Notes.

[2] Hillerdal G (1997). Chylothorax and pseudochylothorax. Eur Respir J.

[3] Al-Gahmi A, Alhuthali M, Alrehaili M, Baltow B, Tashkandi E (2021). Unusual synchronous Association of solid tumors with hematological malignancies in multiple primary cancers: case series and literature review. Case Rep Oncol.

[4] Pan SY, Huang CP, Chen WC (2022). Synchronous/metachronous multiple primary malignancies: review of associated risk factors. Diagnostics (Basel).

[5] Vogt A, Schmid S, Heinimann K, Frick H, Herrmann C, Cerny T, Omlin A (2017). Multiple primary tumours: challenges and approaches, a review. ESMO Open.

[6] Liu S, Wei X, Xiong Y, Mi R, Yin Q (2019). Thirty-two case reports of synchronous hematological malignancy and solid tumor. Turk J Haematol.

[7] Skelton WP 4th, Ali A, Skelton MN (2019). Analysis of overall survival in patients with multiple primary malignancies: a single-center experience. Cureus.

[8] Daly MB, Pal T, Berry MP (2021). Genetic/familial high-risk assessment: breast, ovarian, and pancreatic, version 2.2021, NCCN Clinical Practice Guidelines in Oncology. J Natl Compr Canc Netw.

